# Cholesterol Nanofiber Patches with Sustainable Oil Delivery Eliminate Inflammation in Atopic Skin

**DOI:** 10.1021/acsami.4c09400

**Published:** 2024-07-12

**Authors:** Ewa A. Sroczyk, Aleksandra Tarasiuk, Marcin Talar, Gregory C. Rutledge, Adam Makaro, Zofia Misztal, Maria Wołyniak, Krzysztof Berniak, Maciej Sałaga, Jakub Fichna, Urszula Stachewicz

**Affiliations:** † Faculty of Metals Engineering and Industrial Computer Science, AGH University of Krakow, Al. Mickiewicza 30, 30-059 Krakow, Poland; ‡ Department of Biochemistry, Faculty of Medicine, Medical University of Lodz, Mazowiecka 5, 92-215 Lodz, Poland; § Department of Chemical Engineering, Massachusetts Institute of Technology, Cambridge 02139, Massachusetts, United States

**Keywords:** cholesterol, patches, oil delivery, oil transport, atopic, skin

## Abstract

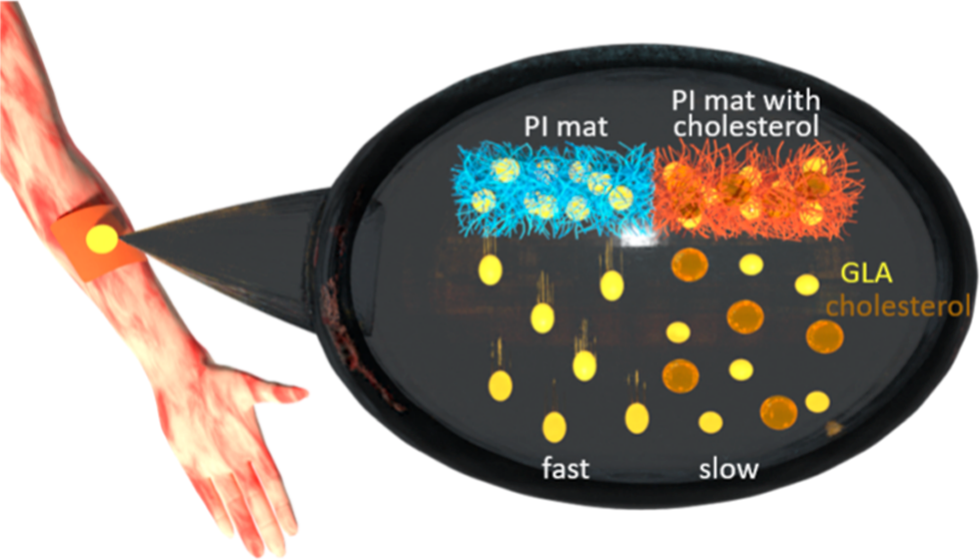

Atopic skin is dry and itchy and lacks integrity. Impaired skin barrier results from altered lipid composition of the skin. A crucial skin lipid, cholesterol, provides flexibility and homeostasis of the cell membranes’ lipid bilayer. Cholesterol-based creams and natural oils, especially blackcurrant seed oil, are beneficial for skin care as they hydrate the skin and improve its integrity. The major atopic symptom, skin dryness, can be overcome by the application of porous patches enhanced with cholesterol and natural oil. The base of the patches is constructed of polyimide (PI) nanofibers with cholesterol coatings and externally added blackcurrant seed oil. The presence of cholesterol in PI mats hinders the passage of oil through the patches to the skin, resulting in sustained and prolonged skin hydration. The theoretical and numerical investigations of oil dynamics in porous mats confirmed the experimental results, showing a prolonged skin hydration effect up to 6 h. Additionally, as demonstrated by in vivo tests on atopic mice, cholesterol patches lower serum immunoglobulin E levels and expression of proinflammatory cytokines in the skin, thereby accelerating skin healing. Our results hold great promise for the long-term application of the patches in atopic dermatitis treatment.

## Introduction

Atopic dermatitis is one of the most common dermatologic diseases, affecting both children and adults. This disease contributes to social withdrawal, depression, and restrictions in sports and leisure activities,^1^ which substantially decrease the quality of life.^2^ Treating this disease poses significant challenges, requiring advancements in both pharmaceuticals and biomedical devices. Moreover, cytokines contribute to inflammation and exacerbation of atopic lesions.^3^ Indeed, atopic patients exhibit higher levels of immunoglobulins E (IgE), which is overproduced as a consequence of inflammatory response and contributes to the prolongation of atopic lesions.^4^ Furthermore, the genetic alterations in cell-mediated immune responses lead to disruption of the stratum corneum (SC), the outermost layer of skin.^5^ Also, atopic patients are deficient in the enzyme delta-6-desaturase which contributes to the conversion of linoleic acid to gamma-linolenic acid (GLA). Importantly, topical application of GLA reduces transepidermal water loss (TEWL) and improves skin integrity.^6^ In SC, cholesterol, free fatty acids, and ceramides are the main lipids;^7^ however, for atopic skin their ratio is disrupted,^8,9^ causing excessive skin desquamation.^9^

Blood level of cholesterol (total cholesterol and low-density lipoprotein cholesterol, LDL) is relatively high in atopic patients,^10,11^ except high-density lipoprotein cholesterol (HDL). In fact, HDL is negatively correlated with the severity of atopic dermatitis.^12^ Importantly, cholesterol makes SC lipid bilayer more flexible,^13^ improving skin homeostasis and resulting in faster skin recovery.^14^ The relationship between cholesterol and atopic dermatitis still remains unclear, yet formulations containing cholesterol effectively treat atopic skin restoring skin barrier and reducing TEWL.^15^ An equimolar mixture of cholesterol, ceramides, and fatty acids applied topically on the skin accelerates skin repair.^16^ Cholesterol has been used only as a constituent of creams for atopic dermatitis applied topically to the skin.

The current methods for atopic skin treatment include avoiding irritating factors and two additional approaches: antimicrobial and ameliorating. Antimicrobial activity is realized by topical application of corticosteroids^17^ and antimicrobial agents in the form of skin patches^18^ or solutions to take a bath in.^19^ Complementary atopic dermatitis therapy requires maintaining skin moisturized and is realized by the application of hydrating formulas such as creams,^20^ emollients,^20^ and natural oils. Natural oils are recommended for atopic patients as they soothe the skin, ameliorate rashes, improve skin barrier integrity, and create an occlusive layer preventing excessive TEWL.^21^ Plant-based oils, such as borage,^22^ evening primrose,^23^ or blackcurrant seed,^24^ are especially beneficial for the skin. Particularly desired is blackcurrant seed oil, which is rich in essential fatty acids, particularly valuable GLA, tocopherols, phytosterols, and triacylglycerols.^25^ Hydrating formulas can be applied either directly to the skin surface or delivered from patches applied on the skin. This textile-based therapy is a well-established method used in the treatment of skin diseases.^26^

To effectively treat atopic dermatitis, a sustained release is desired to provide constant drug concentration and keep the skin hydration level high.^27^ Thus, skin patches are widely used in dermatology because they deliver the desired substances directly to the skin. To reduce atopic skin dryness,^28^ and enhance the healing of the skin, we developed and produced unique electrospun nanofiber patches with electrosprayed cholesterol for direct application on the skin with the addition of blackcurrant seed oil rich in GLA. We show the crucial role of cholesterol in maintaining and restoring the skin barrier, preventing water loss and improving skin hydration levels by working synergistically with oil present in the patch. This novel formulation developed within our research is beneficial for treating atopic skin because it slows the release of oil and simultaneously enhances the delivered oil with cholesterol. The patches can be safely applied for overnight treatment to prolong the curing effect of oils and cholesterol and increase skin moisture. Additionally, in vivo tests on atopic mice demonstrated that these patches lower serum IgE levels and reduce the expression of proinflammatory cytokines in the skin, thereby accelerating skin healing. The promising results of this research suggest that these patches could be a valuable long-term treatment option for atopic dermatitis.

## Results and Discussion

### Characterization of Polyimide (PI) and PI with Cholesterol Mats

**Figure 1:**
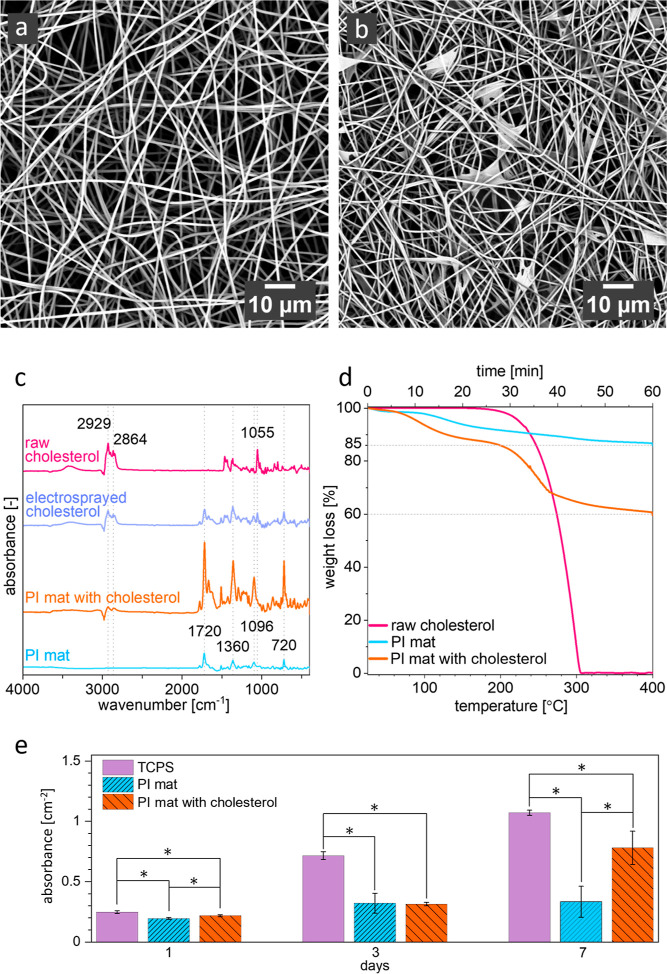
SEM micrographs of (a) PI mat, (b) PI mat with cholesterol, (c) FTIR spectra of the PI mat, PI mat with cholesterol, electrosprayed cholesterol, and raw cholesterol, (d) TGA results of these mats and raw cholesterol, and (e) MTS results indicating proliferation of keratinocytes on PI mat, PI mat with cholesterol, and control substrate—TCPS. *Statistically significant differences (*p* < 0.05) from the nonparametric Kruskal–Wallis test.

PI fibers were successfully electrospun similar to a previous study.^29^ Mats of PI with cholesterol were obtained by simultaneous cholesterol electrospraying and PI electrospinning, uniformly distributing cholesterol between the PI fibers over the whole mat volume; see Figure 1b. The characteristic bands of PI and cholesterol were confirmed with Fourier-transform infrared spectroscopy (FTIR), see Figure 1c and Table S1 in the Supporting Information. Notably, dissolving cholesterol in dimethylacetamide (DMAc) results in an additional peak at 1720 cm^–1^ showing symmetric C=O stretch^30,31^ which comes from residual DMAc presence. Additional peak in electrosprayed cholesterol at 720 cm^–1^ representing methylene chain^32^ suggests increasing number of those and/or their harmonic vibrations.^33^ Increased number of methylene chains in cholesterol results in disordering the SC lipid bilayer and consequently higher fluidity of the membrane.^34^ This effect is desired for the cell membrane integrity and beneficial for atopic skin treatment. Additionally, the thermogravimetric analysis (TGA, see Figure 1d) allows for the estimation of the mass ratio of cholesterol and PI in mats as m_chol_/m_PI_ = 0.42. See the detailed calculations in the Supporting Information. Based on the density of PI ρ_PI_ = 1.43 g cm^–3^^35^ and cholesterol ρ_chol_ = 1.07 g cm^–3^,^36^ the solidity of the PI mat with cholesterol was calculated as 0.07; thus, the cholesterol mass content in the PI mat is 2.5%. For the case in which all pores in the fibrous mat are filled with oil, the mass concentration of cholesterol in oil is 2.7%. Furthermore, PI and PI with cholesterol mats are characterized by a hydrophobic contact angle of 135 ± 1° (Figure S1 in Supporting Information). The beneficial effects of cholesterol are visible in the higher proliferation of keratinocytes on the PI mat with cholesterol compared to that on the PI mat (Figures 1e and S2 in Supporting Information). Cholesterol is a constituent of cell membranes and is likely incorporated into them during keratinocyte growth.^37^ Furthermore, the high absorbance value for the control tissue culture polystyrene (TCPS) sample can be interpreted as keratinocytes’ preference for flat substrate^38^ compared to rough electrospun mats’ surfaces. The biocompatibility of PI mats and PI films has been already confirmed with fibroblasts,^39^ as well as the high stretchability of PI mats of 373%,^39^ indicating the suitability of this material application to the skin. Moreover, scanning electron microscopy (SEM) imaging corroborated the findings from the MTS proliferation assay, demonstrating the growth of cellular populations on both scaffolds (Figure S2a,c for PI mat and Figure S2g,i for PI mat with cholesterol). Furthermore, confocal laser scanning microscopy (CLSM) imaging revealed multiple adhesion sites, characterized by paxillin accumulation, between the cells and the scaffold. Notably, no visible qualitative difference in cellular adhesion was observed between these two material variants.

### Oil Transport in Porous Mats—Theoretical Investigation

The oil transport in the skin patches was investigated via numerical modeling and experimentally. Vertical fluid penetration into porous media can be described by Darcy’s law and Kozeny–Carman equation.^40^ Based on Marmur’s work,^41^ the rate of liquid penetration vertically in short samples is not affected by gravity, thus considering our particular case, the kinetics of oil penetration into electrospun mats can be described with eq 1
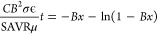
1where *C* is a constant depending on the pore geometry, σ is the surface tension, ϵ is the mat porosity, SAVR is the surface area-to-volume ratio, μ is the dynamic viscosity, *t* is the time, *x* is the distance penetrated by liquid, and *B* is the parameter described with eq 2
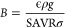
2where ρ is density and *g* is gravity. *C*, called also a degree of true sphericity or shape factor, is the ratio of the surface of a sphere having the same volume as the particle to the surface of this particle.^41,42^ Cholesterol creates coatings between the fibers, substantially changing the shapes of pores into more irregular ones (Figure 1b). PI mat pores exhibit a shape closer to a circle as circular pores are characterized by the shape factor of 1, see Table S2 in the Supporting Information.

### Oil Transport in Porous Mats—Numerical Investigation

**Figure 2:**
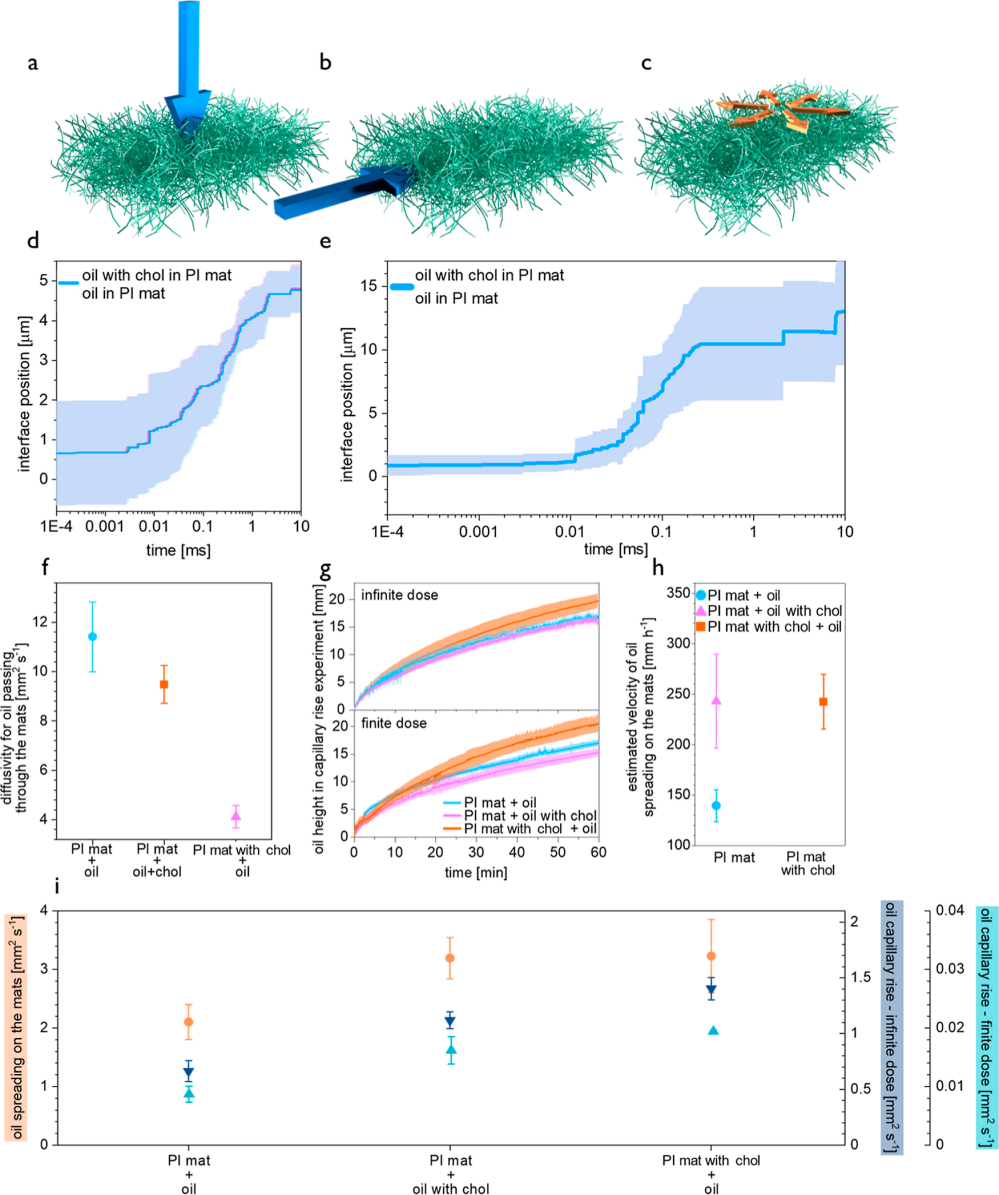
Schematics of (a) oil passing through the mat, (b) oil capillary rise along the mat, and (c) oil spreading on the mat. Position of the air–oil interface in numerical simulation of oil and oil with cholesterol mixture (d) passing through PI mat, (e) transported in the plane of the mat, (f) diffusivity of oil or oil with cholesterol mixture in direction through the mats, (g) dynamics of oil capillary rise along the mats, (h) estimated velocity of oil spreading on the mats, and (i) diffusivity of oil or oil with cholesterol mixture in the porous mats in directions along and on the mats.

In the numerical model, the geometrical parameters of the patches, such as the porosity or pore shapes, were embedded. For these simulations, 2D images of the PI mat were used: a cross-sectional focus ion beam-scanning electron microscopy (FIB-SEM) image of the PI mat as well as an SEM micrograph of the PI mat (Figures S3 and S4 in Supporting Information, respectively). To simplify the simulations, a 2D image of the PI mat cross-section was considered, similar to the previous numerical study of GLA delivery from PI patches to the skin.^39^ In the presented numerical simulations, in one plane, the oil flow is led only by a small fraction of the PI fibers and not supported by surrounding fibers. Here, the 2D simulation does not incorporate the synergistic effect of the adjacent fibers. The pores of the PI mat were initially filled by air, and during the simulation, gradually replaced by either pure oil or oil with cholesterol mixture, see simulation Videos S8, S9, S10, and S11 in Supporting Information. The interface between air and flowing oil was followed, both for the oils passing through PI mats (Figure 2d), and oils transported in the plane of the PI mat (Figure 2e). The most dynamic changes are in the first 1 ms of simulation; see the log scale in *X*-axes in Figure 2d,e. There is no difference in the rate of oil or oil with cholesterol mixture penetration in electrospun PI mats. This phenomenon occurs both for numerical simulations of oil passing through the mat (Figure 2d), as well as experimental results of the transport in the plane of the mat (Figure 2g,h). For the simulation of oil transport in the plane of the mat (Figure 2e), despite a sufficient oil supply, it does not reach the top of the PI mat. The same phenomenon is observed for the capillary rise experiment–both finite and infinite dose; see Figure S5 and Videos S1 and S2 in Supporting Information.

### Oil Transport in Porous Mats—Experimental Investigation

The experimental analysis of oil passage in the patches was conducted in two ways: first, within and through the mats (see Figure 2a and Videos S3, S4 and S5), and second, in the plane of the mats (see Figure 2b and Videos S1 and S2 in Supporting Information; and Figure 2c and Videos S6 and S7 in Supporting Information). Having analyzed the experimental data of oil capillary rise along the mats, the shape factor of the pores of PI mats with cholesterol is 20–70% greater than that of pure PI mats. Apart from the pore shape, the wettability of the mats plays an important role^43,44^ because the oleophilic character of PI (Figure S6 in Supporting Information) promotes oil transport by driving capillary action in the mats. Altered pore shapes of PI with cholesterol mats result in more dynamic penetration into the mat (see the experimental results in Figure 2g, and compare the videos from the experiments of oil capillary rise along the PI mat—Video S1 in Supporting Information, and the PI mat with cholesterol—Video S2 in Supporting Information) and final higher position of oil after 1 h of the capillary rise experiment; see Figure S7 in Supporting Information. Two dosing approaches were applied experimentally to verify if the mats’ pores were capable of imbibing various amounts of oil. Porosity and the pore shape are crucial in fluid transport in mats as it has been indicated in previous studies,^45^ especially pore circularity in controlling directions of oil spreading.^46,47^

### Diffusivity of Electrospun Mats for Oils

To further analyze the oil flow in two geometries of the PI mats with and without cholesterol, the diffusivity of oil or oil with cholesterol mixture in two types of mats was calculated. Generally, the electrospun mats are more diffusive in direction through the mats (Figure 2f) than that in the plane of the mat, such as along (200–1300 times more) or onto the mats (Figure 2i, 1.2–5.4 times more). Yet, PI mats with cholesterol are more diffusive in the plane of the mats (Figure 2i) than through the mat (Figure 2f). This hindering in oil transport through the PI mats with cholesterol stems from the cholesterol presence. Cholesterol deposited between the PI fibers has to be first dissolved by oil, which slows down the oil transport. Yet, in the plane of the mat, this cholesterol in the mats attracts the oil, accelerating the oil transport (Figure 2h), see videos from the experiments of oil spreading on the PI mat (Video S6 in Supporting Information) and the PI mat with cholesterol (Video S7 in Supporting Information). Notably, the form of cholesterol as coatings between the PI fibers is crucial.

**Figure 3:**
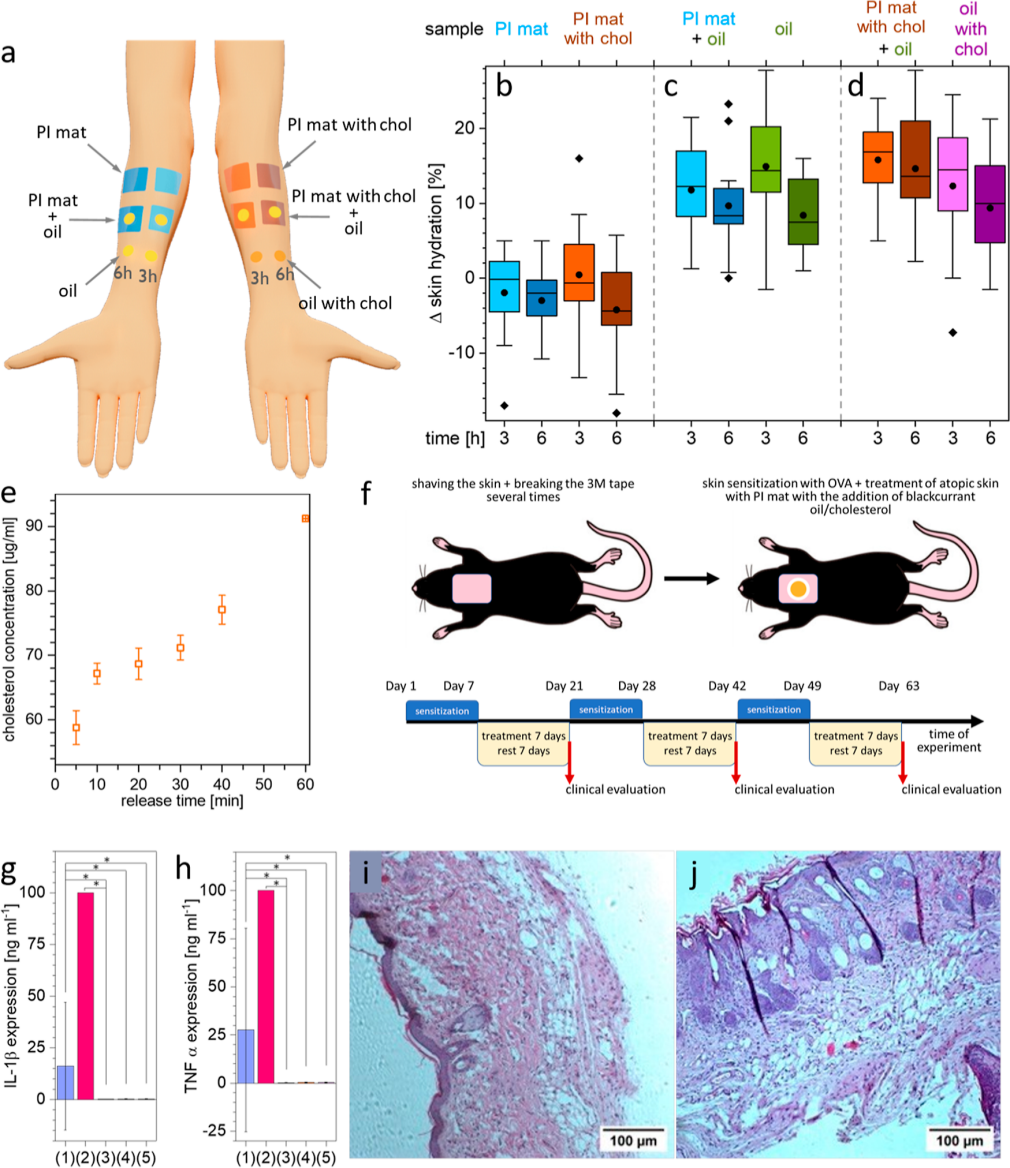
(a) Scheme of experimental arrangement of patches and oils on volunteers’ forearms. Differences in the skin hydration level in 14 volunteers, before and after application of (b) empty mats, (c) PI mats with pure oil and pure oil, (d) PI mats with cholesterol with pure oil, and oil with cholesterol mixture, (e) amount of cholesterol release from PI mats with cholesterol to blackcurrant seed oil, (f) scheme of in vivo experiment on atopic mouse, and (g) expression of IL-1β and (h) TNF-α in OVA-sensitized mouse skin of 10 mice after treatment with patches. *X*-axes legend: (1) control, (2) after sensitization with OVA, (3) after sensitization with OVA and treatment with pure PI mats, (4) after sensitization with OVA and treatment with PI mats with oil, and (5) after sensitization with OVA and treatment with PI mats with cholesterol with oil. *Statistically significant differences (*p* < 0.05) from the nonparametric Kruskal–Wallis test. Pictures of mouse skin sections after (i) sensitization with OVA, (j) sensitization with OVA, and treatment with PI mats with cholesterol with oil.

The difference in diffusivity for pure PI mat, either with oil or oil with a cholesterol mixture, comes from the difference in dynamic viscosity for liquids (Figure S8 in Supporting Information). The dynamic viscosity of pure oil is 56.6 ± 1.4 mPa·s, which provides faster fluid passage than oil with the cholesterol mixture of viscosity of 59.4 ± 0.4 mPa s. Compared to another case, the diffusivity of electrospun poly(vinyl butyral-*co*-vinyl alcohol-*co*-vinyl acetate) (PVB) mats: nano-PVB mats are more diffusive as well as PI mats. However, micro-PVB mats are less diffusive as well as PI mats with cholesterol.^47^ Also, the spreading areas of oil are correlated: oil finally covers a greater area and spreads faster on PI mats with cholesterol, nano-PVB mats, and polycaprolactone (PCL) mats comprising aligned fibers.^46−48^ Yet, oil release from PCL mats is determined by PCL fibers morphology and is less dependent on fibers’ arrangement—PCL mats of porous fibers release a higher fraction of hemp oil than PCL mats of smooth fibers.^46^ Importantly, this diffusivity factor is responsible for more effective skin hydration. Namely, both more diffusive mats are in the plane of the mats: PI mats with cholesterol and nano-PVB mats yield a higher increase in the skin hydration level after 6 h since patch application (Figure 3c,d).

### Skin Hydration by Nanofiber Patches

The skin hydration levels were evaluated before and after the application of mats and patches with oils; see the experimental arrangement in Figure 3a and the example picture in Figure S9 in Supporting Information. In Figure 3b–d, the box plots indicate the changes in Δskin hydration %. Importantly, the variations in Δskin hydration % strongly depend on the initial skin hydration level before the test. Here, the tests were performed on healthy skin having typically higher hydration than the atopic skin; see Figure 3b–d which is indicated in the error bars in Δskin hydration. This stems from the character of the in vivo experiment. The statistically significant differences between the samples used in skin hydration experiments are summarized in Tables S3–S5 in the Supporting Information. Only the first group of control mats (PI mats and PI mats with cholesterol) differs from the two other groups of patches and oil mixtures. Therefore, PI mats with oil as well as PI mats with cholesterol with oil substantially increase the skin hydration, both after 3 and 6 h of application. In Figure 3, the first section (Figure 3b) shows the difference in skin hydration before and after application of empty PI mats, causing the dehydration of the skin. The next section (Figure 3c) contains the results for the PI mat and oil, as well as pure oil application directly on the skin surface. The skin hydration level increased more after 3 h of oil application than after 6 h. That means, the pure oil application, without the mats, improves skin hydration but in a short-time perspective. Similarly, prolonged skin hydration is observed also for PI mats with cholesterol with oil; see Figure 3d. We observe that the oil transport in the patches is strictly related to the skin treatment performance because it determines the delivery rate of healing substances such as GLA and cholesterol. PI patches with blackcurrant seed oil rich in GLA have already been studied and proved to substantially increase skin hydration after 3 h of application and maintain this effect up to 6 h.^39^ Analyzing the increase in the skin hydration level after patch application in this study, both PI mats with oil and PI mats with cholesterol with oil provide moisturization to the skin after 3 and 6 h of application. Topical application of pure oil or oil with cholesterol mixture on the skin without the mats improves skin hydration, but in a short-time perspective, up to 3 h (Figure 3c,d). After this time, the skin hydration effect decays. Yet, the delivery of oil or oil with cholesterol mixture can be prolonged by the usage of electrospun patches. Moreover, cholesterol is gradually released from PI mats with cholesterol to the oil; see Figure 3e. Both PI mats and PI mats with cholesterol provide sustained release of oil and oil with cholesterol mixtures as the oil is released over a longer time than directly putting it on the skin surface.

### Therapeutic Effect of PI Mats with Cholesterol

To test the therapeutic potential of prepared patches in combination with cholesterol and GLA from blackcurrant seed oil, we used a well-established mouse model of atopic dermatitis induced by sustained exposure of skin to OVA.^49^ After three 1 week cycles of treatment with OVA, we observed a significant immune response manifested by increased expression of pro-inflammatory cytokines: interleukin 1β (IL-1β) and TNF-α (Figure 3g,h). We observed that OVA significantly increased the level of IgE in mice, and the opposite effect was seen upon treatment with PI mats, PI mats with oil, and PI mats with cholesterol and oil (below the level of detection; see Figure S10 in Supporting Information). On the histopathological level, a loss of tissue architecture, an increase in the epidermal thickness (10.63 ± 1.99 μm vs 6.61 ± 1.85 μm for OVA and control, respectively), and spongiosis were observed (Figure 3i,j). As shown in Figure 3g,h, the application of PI mats with oil and PI mats with cholesterol and oil reduces the molecular parameters responsible for inflammation in comparison to applying pure PI mats. As shown in Figure 3g,h, the level of IL-1β and TNF-α in the skin decreased to the level below control in mice treated with PI mats with oil and PI mats with cholesterol and oil. Histopathological analysis of skin microphotographs revealed that treatment with PI mats with cholesterol and oil also decreased epidermal thickness (10.43 ± 1.08 and 2.48 ± 0.56 μm, for PI mats with oil and PI mats with cholesterol and oil, respectively) and alleviated the spongiosis of the skin. Our in vivo studies confirm the observations made in the human skin hydration measurements and demonstrate that both PI mats and PI mats with cholesterol not only provide prolonged moisture but also alleviate the severity of the immune response observed in atopic dermatitis. Reduction of pro-inflammatory cytokine expression in the skin shows a topical effect that leads to skin healing. Both IL-1β and TNF-α are cytokines responsible for the recruitment of immune cells to the site of inflammation that in turn leads to tissue damage and therefore the development of symptoms of the disease. The lowering of total IgE in the serum of mice treated with mats demonstrates that a systemic therapeutic effect also was achieved. GLA and cholesterol from PI mats with cholesterol with oil eliminate the inflammation and maintain atopic skin hydrated, see Figure S10. Importantly, the numerical simulations and experiments indicated that adding cholesterol to patches promotes oils spreading and slows the oils transport through the patches, giving the prolongated skin hydration effects verified by the tests on human skin. Together those results hold promise for future applications of PI mats combined with cholesterol and GLA from blackcurrant seed oil in the therapy of atopic dermatitis.

## Conclusions

Within our study, we fabricated nanofiber PI mats with cholesterol and added blackcurrant seed oil to provide a complementary system for atopic skin treatment. Patches rich in cholesterol and gamma linoleic acid (GLA) allow long-term skin hydration, which is more effective than putting oil directly on the skin surface. This effect is implemented by the complex architecture of PI mats with cholesterol, where the cholesterol coatings are deposited between PI nanofibers. Consequently, oil is transported slower through the PI mat with cholesterol but faster in the plane of this mat, promoting oil spreading, which was confirmed by a set of experiments and the numerical study. In vivo study on atopic mice proves therapeutic effects of cholesterol patches by a significant reduction of IL-1β and TNF-α expression. Moreover, cholesterol plays a crucial role in maintaining and restoring the skin barrier, which prevents water loss. Incorporating cholesterol has shown significant improvements in skin hydration levels and the overall severity of atopic dermatitis symptoms. Furthermore, cholesterol works synergistically with other key lipids present in oils, enhancing its effectiveness in managing skin condition. Overall, the incorporation of cholesterol into PI mats not only prolongs hydration but also provides significant anti-inflammatory benefits, making these mats promising candidates for the long-term treatment of atopic dermatitis.

## Methods

### Samples Fabrication

PI mats and PI films were prepared similar to the previous study.^39^ PI P84 granulates obtained from Ensinger Sintimid GmbH, Austria, were dried over 4 h at 50 °C (drying oven, POL-ECO Aparatura, Poland) prior to preparing the 18% PI solution in dimethyl sulfoxide (Avantor, Poland) and DMAc (Avantor, Poland) mixture in the mass ratio 3:7. The solvents (analytical standard) were purchased from Avantor, Poland. PI mat was electrospun using a 21-gauge stainless-steel needle kept at a 15 cm distance to the collector covered with wax paper in the equipment from IME Technologies (The Netherlands). The high voltage of 18 kV was applied to the nozzle, and the PI solution flow rate was set to 0.30 mL h^–1^.

Cholesterol (Acros Organics, The Netherlands) was dissolved in DMAc to achieve a 5% solution. PI mats with cholesterol were obtained by simultaneous conduction of PI electrospinning and cholesterol electrospray using a two-nozzle system, see Figure S11 in Supporting Information. Electrospraying parameters were 15 cm distance of the nozzle to the substrate and 0.80 mL of h^–1^ flow rate at the same environmental conditions. To distribute evenly both cholesterol and PI fibers, the collector rotated with a velocity of 10 rpm, and both needles translated parallel to the axis of the collector with a velocity of 20 mm s^–1^ over a 15 cm distance. The fabrication of both pure PI mat and PI mat with cholesterol was performed over 1.5 h at RH = 50–60% and *T* = 24 °C.

PI films were prepared out of the same PI solution on a glass slide using a spin-coater (L2001A v.3, Ossila, UK) at 6000 rpm for 60 s under the same environmental conditions.

### Characterization of the Samples

The samples were investigated using SEM (Thermo Fisher Scientific Phenom ProX G6, USA) after coating them with a gold 8 nm layer using a Q150RS sputter coater (Quorum Technologies, UK). The samples were imaged with a 5.3 kV accelerating voltage, 40 μA, and working distance in the range 7.7–8.1 mm.

FTIR was performed with a Nicolet iS5 FT-IR spectrometer (Thermo Fisher Scientific, USA) in the range 4000–400 cm^–1^. Spectra were taken with a resolution of 4 cm^–1^ and were averaged over 64 scans in a reflection mode using a diamond crystal. FTIR spectra were processed with Omnic software (Thermo Fisher Scientific, USA).

The contact angles of water on the mats (Figure S1) were evaluated using deionized water drops (3 μL) placed on the samples. The images of water droplets were taken after 10 s at RH = 40% and *T* = 22 °C using a Canon EOS 700D camera with an EF-S 60 mm f/2.8 Macro USM zoom lens (Japan). The contact angles were determined from an average of 10 drops analyzed in ImageJ (J1.53v, NIH, USA).

PI film contact angles of blackcurrant seed oil (Au Natural Organics, USA) and its mixtures with cholesterol (Acros Organics, The Netherlands) (Figure S6) were evaluated with a Nikon D5300 camera with AF-S Micro Nikkor 105 mm f/2.8 lens (Japan). The serial dilutions of cholesterol in oil were prepared between 0 and 3% with step 0.5. The environmental conditions were RH = 25% and *T* = 20 °C.

Cholesterol content in PI with cholesterol mat was evaluated by TGA on a Thermogravimetric Analyzer Discovery (TA Instruments, USA) with a heating rate of 10 °C min^–1^ from 25 to 400 °C. The experiments were carried out under a nitrogen flow of 50 mL min^–1^. The 4–8 mg of samples were put on platinum high-temperature pans. From the TGA results, we evaluated the mass of cholesterol in PI mats with cholesterol. Based on the solidity formula
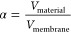
3where α is the solidity, *V*_material_ is the volume of material (e.g., PI fibers), and *V*_membrane_ is the mat volume.

### Oil with Cholesterol Mixture Characterization

We prepared the serial dilutions of cholesterol in blackcurrant seed oil (Au Natural Organics, USA) between 0 and 3% with step 0.5. The densities of those were measured using VWR Signature Ergonomic High-Performance Pipettor (model VE200) calibrated according to the EN ISO 8655 standard and balance VWR-64B. The environmental conditions were a temperature of 20 °C and RH = 25%.

Rheological characterization was performed using a Rheometer AR2000 (TA Instruments, USA), with each measurement of 700 μL of the mixture, each sample in triplicate. The geometry used was a standard steel parallel plate of 25 mm diameter. Each experiment was carried out in the range 0–80 Pa of shear stress in the linear ramp mode taking nine measuring points. Gap tolerance was 5%.

The surface tension of the mixtures was measured with a contact angle apparatus (Rame-Hart, USA) by pendant drop shape analysis. The shape of the drop hanging from a needle is determined from the balance of forces, which includes the surface tension of the liquid being investigated. The surface tension was measured using DropImage software (Rame-Hart, USA).

### Oil Transport in Porous Mats

Blackcurrant seed oil (Au Natural Organics, USA) and this oil with 2.7% of cholesterol (Acros Organics, The Netherlands) mixture transport in PI mats and PI mats with cholesterol were investigated. The mats were placed on a glass slide and observed from the top and the bottom—see Figure S12. The VWR Signature Ergonomic High-Performance Pipettor (model VE200) pipet was kept at a fixed distance of 5 mm from the investigated mats. The pipetted volume of oil or oil with cholesterol mixture was 20 μL, and the mats were cut into 3 × 3 cm squares.

Oil or oil with cholesterol mixture passing through the PI mats and PI mats with cholesterol was recorded with a high-speed camera CR14–1.0 (Krontech, Canada) using an EF-S 60 mm f/2.8 Macro USM zoom lens at a speed of 1000 fps. The adequate setup allowed for catching the moment of the droplet touching the surface of the mat, as well as this droplet having passed the mat and touching the glass slide—see Videos S3, S4, and S5. The time of oil passing the mats was taken from the recorded videos—see Figure S13. The thicknesses of the mats were measured with light microscope imaging in the *z*-direction (Axio Imager M1m, ZEISS, Germany).

Furthermore, the spreading of oil and oil with cholesterol mixture on PI mats and PI mats with cholesterol was investigated with a Canon EOS 700D camera with an EF-S 60 mm f/2.8 Macro USM zoom lens (Japan). The series of photographs were analyzed with custom macros for ImageJ software (J1.53v, NIH, USA).

Oil imbibition experiment: PI mats and PI mats with cholesterol were rolled around 0.8 mm copper wires to keep their flat shape during experiments—see Figure S5. The bottom edge of the mat was soaked in oil or a cholesterol mixture. The oil rise was observed with a Nikon D5300 camera with an AF-S Micro Nikkor 105 mm f/2.8 lens (Japan) for a finite dose experiment, where the mat was only dipped in oil and immediately taken out from oil. On the infinite dose experiment, oil was constantly provided over the whole experiment (over 1 h). For an infinite dose experiment, a Canon EOS 700D camera with an EF-S 60 mm f/2.8 Macro USM zoom lens (Japan) was used. The series of photographs were analyzed with custom macros for ImageJ software (J1.53v, NIH, USA).

### Cholesterol Release from PI with Cholesterol Mats

Four PI mats with cholesterol of size 3 × 3 cm were put into beakers filled with 3 mL of blackcurrant seed oil (Etja, Poland) and shaken with a velocity of 120 rpm at 22 °C (Shaker IKA KS 3000 IC Control, Germany). The release of cholesterol from these mats to the oil was investigated over time after 5, 10, 20, 30, 40, and 60 min. The amount of released cholesterol was measured with an UV/vis spectrophotometer UV7 (Metler Toledo, Belgium) for a wavelength of 328 nm. The oil with released cholesterol was poured into testing cuvettes for measurement and then put back into the beaker for the ongoing experiment. The concentration of released cholesterol was calculated from a calibration curve for 328 nm, see Figure S14.

### Numerical Simulation of Multiphase Flows

For multiphase flows, the COMSOL Multiphysics program (version 5.6, COMSOL Inc., Sweden) was used. PI mat was observed from two different angles. A cross-section image of the PI mat was obtained by focus ion beam SEM (FIB-SEM, see Figure S3), then postprocessed and imported to the COMSOL Multiphysics program. For the top-view simulation, an SEM image of the PI mat was used (see Figure S4). PI mat pores were initially filled with air. The oil reservoir provided blackcurrant seed oil with a density of 847.8 kg m^–3^, dynamic viscosity of 0.0566 Pa s, and surface tension of 0.0325 N m^–1^. Blackcurrant seed oil with 2.7% of cholesterol mixture was modeled as pure oil with a different dynamic viscosity value of 0.0594 Pa s. All these parameters are experimentally gathered data. Periodic conditions on the left and right edges, as well as gravity force, were applied in both simulations. According to computational resources limitation, a contact angle between the oil and PI fiber of 15° was used for the simulation. For the surface of the fibers, the Navier slip condition was applied. The thickness of the interface between oil and air was determined as 0.1 nm. The transport of the fluid interface separating oil and air is given by^50,51^
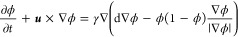
4where ϕ is the phase (denoted as 0 or 1), ***u*** is the velocity field, *d* is the interface thickness, and γ is the parameter determining the amount of reinitialization. Mass and momentum transport for fluids incorporating capillary effects can be described with the Navier–Stokes equation
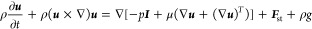
5where ***I*** is the identity tensor, *T* is the matrix transposition, and ***F***_st_ is the surface tension force acting on interface between oil and air calculated from
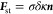
6where δ is the Dirac delta which is nonzero only at the fluid interface, κ is the curvature, ***n*** is the interface normal, and α is the contact angle. Wetted wall coupling feature adds the following term as boundary force
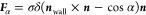
7

For the simulation of oil passage through PI mats, a cross-sectional image was binarized and used for simulation; see Figure S3. The created mesh consisted of 60,821 triangles of size in the range 0.02–0.4 μm built with a maximum growth rate of 1.1, curvature factor of 0.5, minimum orthogonal quality of 0.5491, and average quality of 0.8635. The model dimensions are 15.74 × 6.49 μm (PI mat: 5.45 μm, oil reservoir: 1 μm). The optimal step size was Δ*t* = 0.0001 ms in the range 0–3 ms, Δ*t* = 0.1 ms in the range 3–10 ms.

For the simulation of oil transport in the plane of the mat, an SEM micrograph of the PI mat was used and postprocessed, as depicted in Figure S4. This mesh consisted of 43 709 triangles. Triangles size range, maximum growth rate, curvature factor, and timesteps were kept the same as for the simulation of oil passing through the PI mat. Mesh for simulation of oil transport in the plane of the mat was built with a minimum orthogonal quality of 0.5257 and an average quality of 0.8858. The model dimensions are 18 × 38 μm in total (PI mat: 32 μm, oil reservoir: 6 μm).

### Biocompatibility Assessment

Biocompatibility of PI mats and PI mats with cholesterol was evaluated with immortalized keratinocytes from adult human skin (HaCaT) at 2 × 10^4^ cells per well in 24-well plates. The experiment was conducted over 7 days using Dulbecco’s Modified Eagle Medium (Thermo Fisher Scientific, USA) supplemented with bovine serum (10%), antibiotics (penicillin/streptomycin, 2%), amino acids (1%), and l-glutamine (1%, Sigma-Aldrich, UK). The cell culture was conducted under standard conditions (*T* = 37 °C, RH = 90%, and 5% CO_2_ at atmospheric pressure). TCPS was used as a positive control. The cell culture medium was changed after 3 days, and the condition of the cells was checked after 1, 3, and 7 days.

The cells’ proliferation was assessed by the colorimetric measurement of MTS assay (CellTiter 96 Aqueous One Solution Cell Proliferation Assay, Promega, USA), for each time point triplicate. First, the cell culture medium was discarded, and then 80 μL of the reagent and 400 μL of the fresh cell culture medium were added and incubated over 4 h at standard cell culture conditions. Next, 100 μL of the reacted solution was transferred to a new 96-well plate in four repetitions. In the end, the quantity of the produced formazan was measured quantitatively at a 490 nm wavelength with a spectrophotometer (Promega GloMax Discover Plate Reader, USA). The raw absorbance data were recalculated to the corresponding sample surface area.

For SEM imaging, the cell culture medium was removed at each measuring point. First, the cells were washed with phosphate-buffered saline (PBS) and fixed with paraformaldehyde solution (4%, Sigma-Aldrich, UK) for 15 min at 23 °C. Then, the samples were dehydrated in a series of ethanol solutions (50, 70, 96, and ∼99.9%, Avantor, Poland)—three times in each concentration solution for 3 min. Finally, the samples were coated with 8 nm of gold and imaged as in the microscopy analysis section. The images were processed using ImageJ (J1.53v, NIH, USA).

CLSM was used for cells imaging. First, the samples were fixed with paraformaldehyde solution (4%, Merck, UK) for 15 min at 23 °C. Next, the cells were permeabilized at 23 °C with 0.1% triton X-100 (Merck, UK) in PBS solution for 5 min. Then, the samples were incubated in blocking solution (3% of bovine serum albumin, BSA, Merck, UK, in PBS) for 60 min at 23 °C. For staining actin filaments, cells were incubated for 60 min at 23 °C with Alexa Fluor 488 Phalloidin (Thermo Fisher Scientific, USA). For staining focal adhesion sites, the samples were incubated for 60 min at 23 °C with primary rabbit Anti-Paxillin antibody (ab32084, Abcam, UK) and then followed by incubation with secondary Alexa Fluor 555 goat antirabbit antibody (A-21428, Thermo Fisher Scientific, USA) for 60 min at 23 °C. Nuclear DNA was stained with 4′,6-diamidino-2-phenylindole (DAPI, Merck, UK) at 23 °C for 10 min. Such prepared samples were imaged with a Zeiss LSM 900 confocal microscope (Zeiss, Germany) using a lens Plan-Apochromat 40*x*/1.3 Oil. The images were acquired in the sequential mode by using ZEN 3.1 software (Zeiss, Germany) and processed with ImageJ software (J1.53v, NIH, USA). For excitation: 561, 488, and 405 nm laser lines were used, emission detection bands were set to 545–700 nm for Alexa Fluor 555, 500–550 nm for Alexa Fluor 488 coupled with Phalloidin, and 400–570 nm for DAPI. Electrospun fibers were observed in the transmission light channel.

### Skin Hydration Tests

Experiments on volunteers were performed according to the guidelines for cosmetic product testing on humans with respect to the Council Directive (76/768/EEC) and World Medical Association Declaration of Helsinki (1964–1975–1983–1989–1996). 14 volunteers of skin types I–IV, both female and male, aged 26–42, with healthy skin, participated in the experiment. PI mats and PI mats with cholesterol were cut into 3 × 3 cm squares and mounted onto forearms with medical tape on the edges, see Figure S9.

20 μL of blackcurrant seed oil (Au Natural Organics, USA) was pipetted onto PI mats, PI mats with cholesterol, and also alone directly on the skin surface. Also, an oil-cholesterol mixture was put directly on the skin surface. A corneometer (Hydro Pen H10, Medelink, Canada) was used to measure the skin hydration level before and after the application of mats, patches, or oils in five repetitions in each testing place. The increase in skin hydration was calculated as a simple difference, with deviation recalculated as the standard uncertainty.

### In Vivo Tests on Mice

#### Animals Housing

Experimentally naive male C57BL/6 mice (Animal House at Mossakowski Medical Research Institute, Polish Academy of Sciences, Warsaw) weighing 20–25 g were used. Animals were housed at a constant temperature (20–22 °C), under a 12 h light/dark cycle in sawdust-lined plastic cages. Chow pellets and tap water were provided ad libitum. All animal protocols were approved by the Medical University of Lodz Animal Care Committee (protocol no. 49/LB216/2021) and complied with the European Communities Council Directive of September 22, 2010 the EU (2010/63/EU). All efforts were made to minimize animal suffering and reduce the number of animals used. Animals were divided into five groups (*n* = 10): healthy mice (1); atopic mice (2); atopic mice treated with PI mats (3); atopic mice treated with PI mats + blackcurrant seed oil (Etja, Poland) (4), and atopic mice treated with PI mats with cholesterol + blackcurrant seed oil (Etja, Poland) (5).

#### OVA-Induced Atopic Dermatitis Mouse Model

A mouse model of atopic dermatitis was induced by repeated epicutaneous sensitization of tape-stripped skin with OVA. Briefly, the back skin of mice was shaved, and tape was stripped six times with 3 M tape, mimicking skin injury inflicted by scratching in atopic patients. OVA (100 μg, Merck Life Science, Germany) in 100 μL of normal saline or 100 μL of normal saline was placed on a 1 × 1 cm mat of sterile gauze, which was mounted onto the mouse skin and protected from detachment. This step prevented mice from licking the sensitized skin. Each mouse from groups 2–5 had a total of three 1 week exposures to OVA at the same site, separated from each other by 2 week intervals.

#### Treatment of Atopic Dermatitis with PI Mats

After being exposed to OVA, mice were treated accordingly with or without the respective mats for 7 days. The remaining time until the next OVA exposure was treated as the rest time. The whole procedure lasted 63 days.

#### Blood Serum and Tissue Collection

Following deep anesthesia, blood samples were collected into 2 mL Eppendorf tubes, and the serum was obtained according to an in-house protocol. Vials with serum were immediately frozen at −80 °C until further use. Following euthanasia, the pieces of skin from each animal were removed and immediately frozen in liquid nitrogen and kept at a temperature of 80 °C until needed.

#### Histopathological Analysis

Tissue samples from the skin were fixed in 4% buffered formalin, pH 7.2, for 24 h before being processed automatically in a tissue processor. Paraffin-embedded tissue samples were sectioned, mounted on slides, and stained with hematoxylin and eosin (H&E) according to the normal protocol. An Axio Imager A2 microscope (Carl Zeiss, Germany) was used to examine the samples. Photographs were taken using a digital imaging system consisting of a digital camera (Axiocam 506 color, Carl Zeiss, Germany) and image analysis software (Zen 2.5 blue edition, Carl Zeiss, Germany). A pathologist who was not aware of the experimental protocol performed the morphological analyses.

The thickness of the epidermis was calculated as micrometers (μm) digitally by using image analysis software (Zen 2.5 blue edition, Carl Zeiss, Germany). The epidermis was measured eight times from the free margin of skin to the dermal papillae and epidermal rete ridge. The mean and median, with standard deviation values of epidermis, were calculated using Microsoft Excel (Microsoft Corporation, Redmond, Washington). The mean (μm) ± SD values were presented as epidermal thickness.^52^

#### Determination of Mouse Total IgE, Serum anti-OVA IgE, and IgG Antibodies

Mouse total IgE, serum anti-OVA IgE, and IgG antibodies were determined in mouse blood serum using assay kits in accordance with the manufacturer’s protocol (#3005, #3010, #3011; Chondrex, Inc., USA).

#### RNA Isolation, Reverse Transcription, and qPCR

Briefly, total RNA was isolated from each mouse skin sample in accordance with the manufacturer’s protocol using Total RNA Mini Plus kit (A&A Biotechnology, Poland). RNA was eluted from ion ex-change columns by diethyl pyrocarbonate-treated water. The purity and quantity of isolated RNA were estimated using a Colibri Microvolume spectrophotometer (Biocompare, USA). Total RNA was transcribed to cDNA with the Maxima First Strand cDNA Synthesis Kit for RT-qPCR (Thermo Fisher Scientific, USA), in accordance with the manufacturer’s protocol. mRNA expression of IL1β and TNFα in relation to β-actin was measured in duplicate using the following equation: 2 – Δ*C*_t_ × 1000, where *C*_t_ represents a threshold cycle value in the PCR.

#### Statistical Analyses

The statistical analyses were performed using Statistica (13.3, StatSoft, Inc., USA) at the significance level *p* = 0.05. The Shapiro–Wilk test was used for the evaluation of data normality. Also, ANOVA followed by the Tukey posthoc test for diffusivity data and Fisher’s posthoc test for skin hydration data were performed. For MTS data and antibodies concentration, a nonparametric Kruskal–Wallis test was applied.

## Supporting Information Available

Water contact angles on PI mats and PI mats with cholesterol; SEM images of keratinocytes on PI and PI mat with cholesterol after 1, 3, and 7 days of incubation and CLSM images of keratinocytes on PI and PI mat with cholesterol in the seventh day of incubation; computational domain for numerical simulation of oil flow through the PI mat; computational domain for numerical simulation of oil transport in the plane of PI mat; experimental setup of the capillary rise experiment: example pictures of oil capillary rise along PI mats—finite dose and infinite dose; wettability of PI films with oil and oil with 3% of cholesterol; final oil position in PI mats and PI mats with cholesterol after 1 h of the capillary rise experiment; dynamic viscosity of oil with cholesterol mixtures; representative image from skin hydration tests after patch application; total IgE levels in the mouse sera utilizing Chondrex Elisa KIT; schematics of PI mats with cholesterol fabrication; experimental setup of oil in mats transport investigation; time of oils passing through the mats–experimental data; calibration curve of cholesterol in blackcurrant seed oil UV–vis absorbance; FTIR bands for raw cholesterol, electrosprayed cholesterol, PI mat, and PI mat with cholesterol; shape factor values from fitting the experimental data of capillary rise; and statistically significant differences for data from the skin hydration test (PDF)
Oil capillary rise along PI mat–infinite dose (AVI)
Oil capillary rise along PI mat with cholesterol–infinite dose (AVI)
Oil passing through PI mat (AVI)
Oil passing through PI mat with cholesterol (AVI)
Oil with cholesterol mixture passing through PI mat (AVI)
Oil spreading on PI mat (AVI)
Oil spreading on PI mat with cholesterol (AVI)
PI mat pores filling with oil for numerical simulation of pure oil passing through PI mat (AVI)
PI mat pores filling with oil for numerical simulation of oil with cholesterol mixture passing through PI mat (AVI)
PI mat pores filling with oil for numerical simulation of pure oil transported in the plane of the mat (AVI)
PI mat pores filling with oil for numerical simulation of oil with cholesterol mixture transported in the plane of the mat (AVI)

## Supplementary Material

am4c09400_si_001.pdf

am4c09400_si_002.avi

am4c09400_si_003.avi

am4c09400_si_004.avi

am4c09400_si_005.avi

am4c09400_si_006.avi

am4c09400_si_007.avi

am4c09400_si_008.avi

am4c09400_si_009.avi

am4c09400_si_010.avi

am4c09400_si_011.avi

am4c09400_si_012.avi
