# Supplementary material for: Cholesterol Nanofiber Patches with Sustainable Oil Delivery Eliminate Inflammation in Atopic Skin
Source: ACS Appl Mater Interfaces. 2024 Jul 12;16(29):37783–94. doi: 10.1021/acsami.4c09400 (PMC11284794; doi:10.1021/acsami.4c09400)
Supplement: Supplementary file 1 — am4c09400_si_001.pdf [file am4c09400_si_001.pdf]

Supporting Information

Cholesterol nanofiber patches with sustainable oil delivery eliminate inflammation in atopic skin

Ewa A. Sroczyk[a, c], Aleksandra Tarasiuk[b], Marcin Talar[b], Gregory C. Rutledge[c], Adam Makaro[b], Zofia Misztal[b], Maria Wołyniak[b], Krzysztof Berniak[a], Maciej Sałaga[b]*, Jakub Fichna[b]*, Urszula Stachewicz[a, *]

[a] Faculty of Metals Engineering and Industrial Computer Science, AGH University of Krakow, Al. Mickiewicza 30, 30-059 Krakow, Poland
[b] Department of Biochemistry, Faculty of Medicine, Medical University of Lodz, Mazowiecka 5, 92-215 Lodz, Poland
[c] Department of Chemical Engineering, Massachusetts Institute of Technology, Cambridge, MA 02139, United States
* corresponding authors emails: US: ustachew@agh.edu.pl, JF: jakub.fichna@umed.lodz.pl, MS: maciej.salaga@umed.lodz.pl

This SI file includes:

Figure S1. Pictures of water contact angles on PI mats and PI mats with cholesterol.
Figure S2. SEM images of keratinocytes on PI and PI mat with cholesterol after 1, 3, and 7 days of incubation and CLSM images of keratinocytes on PI and PI mat with cholesterol in the 7th day of incubation.
Figure S3. Computational domain for numerical simulation of oil flow through the PI mat.
Figure S4. Computational domain for numerical simulation of oil transport in the plane of PI mat.
Figure S5. Experimental setup of capillary rise experiment: example pictures of oil capillary rise along PI mats (a) finite dose, (b) infinite dose.
Figure S6. Wettability of PI films with oil and oil with 3% of cholesterol.
Figure S7. Final oil position in PI mats and PI mats with cholesterol after 1 h of capillary rise experiment.
Figure S8. Dynamic viscosity of oil with cholesterol mixtures.
Figure S9. Representative image from skin hydration tests after patch application.
Figure S10. Total IgE levels in the mouse sera utilizing Chondrex Elisa KIT.
Figure S11. Schematics of PI mats with cholesterol fabrication.
Figure S12. Experimental setup of oil in mats transport investigation: (a) front view, (b) side view.
Figure S13. Time of oils passing through the mats – experimental data.
Figure S14. Calibration curve of cholesterol in blackcurrant seed oil UV-vis absorbance

Tables S1 to S5
Table S1. FTIR bands for raw cholesterol, electrosprayed cholesterol, PI mat, and PI mat with cholesterol.
Table S2. Shape factor values from fitting the experimental data of capillary rise to Eq. 1.
Table S3-S5. Statistically significant differences for data from skin hydration test.

Legends for Videos S1 to S11:

Video S1. Video of oil capillary rise along PI mat – infinite dose.

Video S2. Video of oil capillary rise along PI mat with cholesterol – infinite dose.

Video S3. Video of oil passing through PI mat (bottom view).

Video S4. Video of oil passing through PI mat with cholesterol (bottom view).

Video S5. Video of oil with cholesterol mixture passing through PI mat (bottom view).

Video S6. Video of oil spreading on PI mat.

Video S7. Video of oil spreading on PI mat with cholesterol.

Video S8. Video of PI mat pores filling with oil for numerical simulation of pure oil passing through PI mat.

Video S9. Video of PI mat pores filling with oil for numerical simulation of oil with cholesterol mixture passing through PI mat.

Video S10. Video of PI mat pores filling with oil for numerical simulation of pure oil transported in the plane of the mat.

Video S11. Video of PI mat pores filling with oil for numerical simulation of oil with cholesterol mixture transported in the plane of the mat.

SI References

PI mats and PI with cholesterol mats characterization

The addition of cholesterol to PI mats does not alter their hydrophobic character as pure PI mat indicates a similar contact angle of 134 ± 2° [1], see Figure S1.

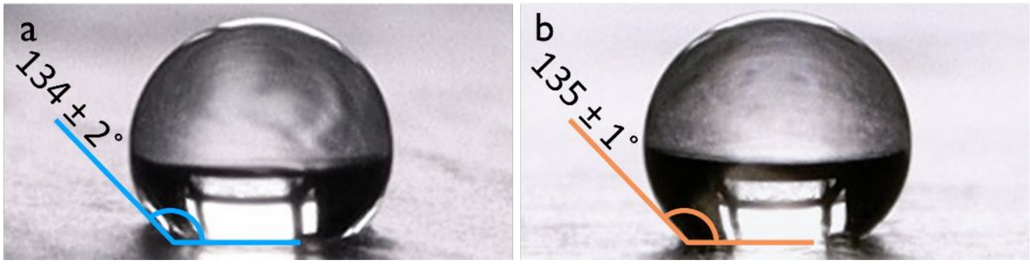

Figure S1. The example of water droplets on (a) PI mat, and (b) PI with cholesterol mat.

We proved the biocompatibility of PI mats and PI mats with cholesterol with HaCaT cells. On the 1st and 3rd day of cell culture, keratinocytes were growing and proliferating at a similar pace on both these mats, see Figure S2. On the 7th day of incubation, keratinocytes created a thick monolayer, accumulating into focal adhesion points marked with arrows in Figure S2d, j.

PI to cholesterol mass ratio calculation

The PI to cholesterol ratio has been calculated based on the TGA results as well as the previous data of PI mat porosity of 95.6% [1]. After heating, the PI mass (m_{PI}) is 85% of the initial mass of the PI mat ($m_{PI\ total}$, see blue curve on Figure 1d) and can be described with the following equation:

$$m_{PI} + m_{PI\uparrow} = m_{PI\ total} \qquad \text{Eq. 8}$$

Due to 15% mass loss coming from the solvents' evaporation ($m_{PI\uparrow}$), one can write the following formula:

$$m_{PI\uparrow} = 0.15 m_{PI\ total} \qquad \text{Eq. 9}$$

Assuming the same mass loss due to solvents' evaporation (15%) in the PI mat with cholesterol as well as total degradation of cholesterol in 304ºC (pink curve on Figure 1d), the mass loss of PI mat with cholesterol in 400ºC is 40% (orange curve on Figure 1d) which can be described as:

$$\frac{m_{PI\uparrow} + m_{chol}}{m_{PI}} = \frac{40}{60} \qquad \text{Eq. 10}$$

The equations 8-10 lead to the mass ratio m_{chol} : $m_{PI\ total}$ = 0.4167.

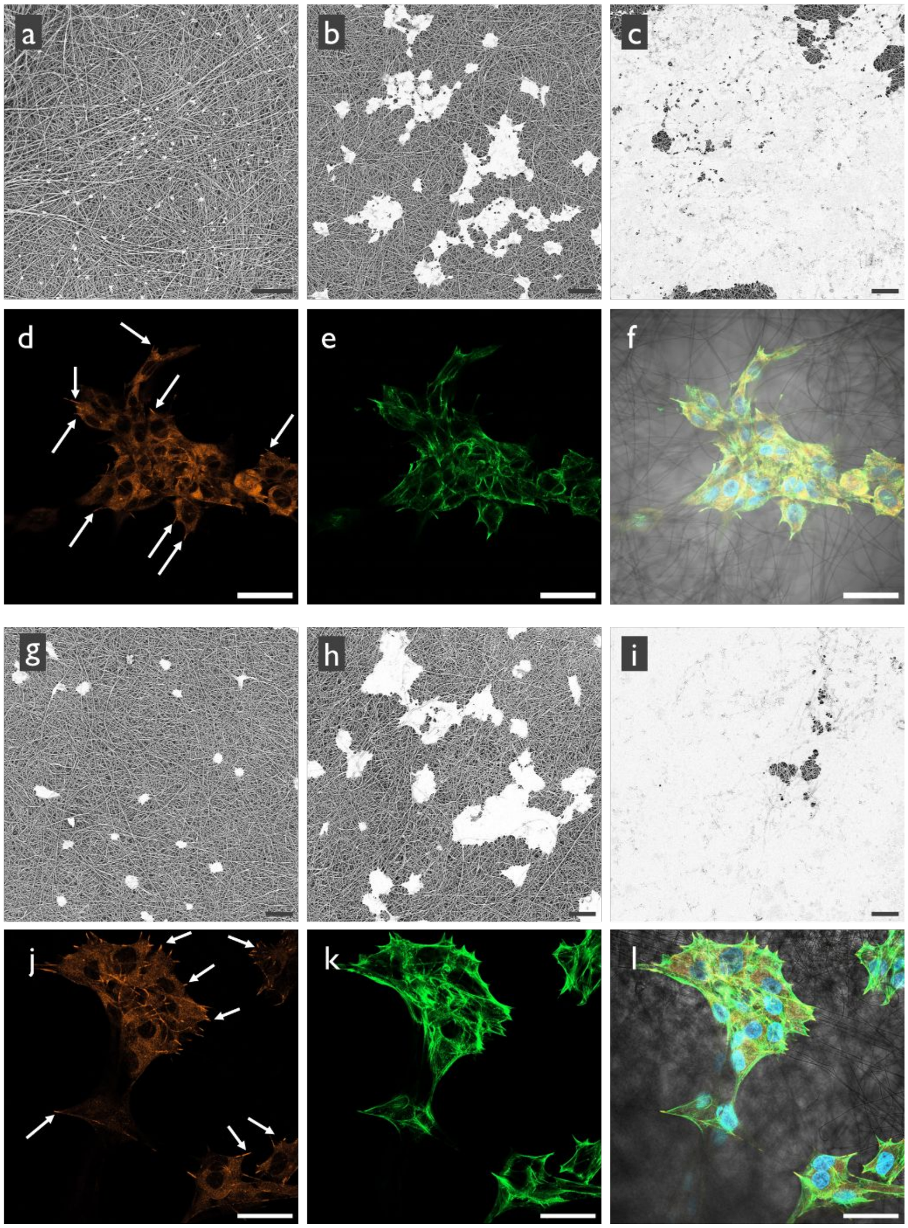

Figure S2. SEM micrographs of keratinocytes on PI mat after (a) 1, (b) 3, (c) 7 days of incubation; CLSM images of keratinocytes in the 7th day of incubation: (d) focal adhesion sites (paxillin), (e) actin filaments, (f) merged image with additional channels of cells nuclei and transmission view of the sample. SEM micrographs of the cells on PI mats with cholesterol after (g) 1, (h) 3, (i) 7 days of incubation; CLSM imaging of those in the 7th day of cells growth: (j) focal adhesion sites, (k) actin filaments, (l) merged image with additional channels of cells nuclei and transmission view of the sample. In all CLSM images paxillin were stained with Alexa Fluor 555 (orange), actin filaments with Alexa Fluor 488 Phalloidin (green), and nuclei with DAPI (blue). The scale bar of the images is 50 μm.

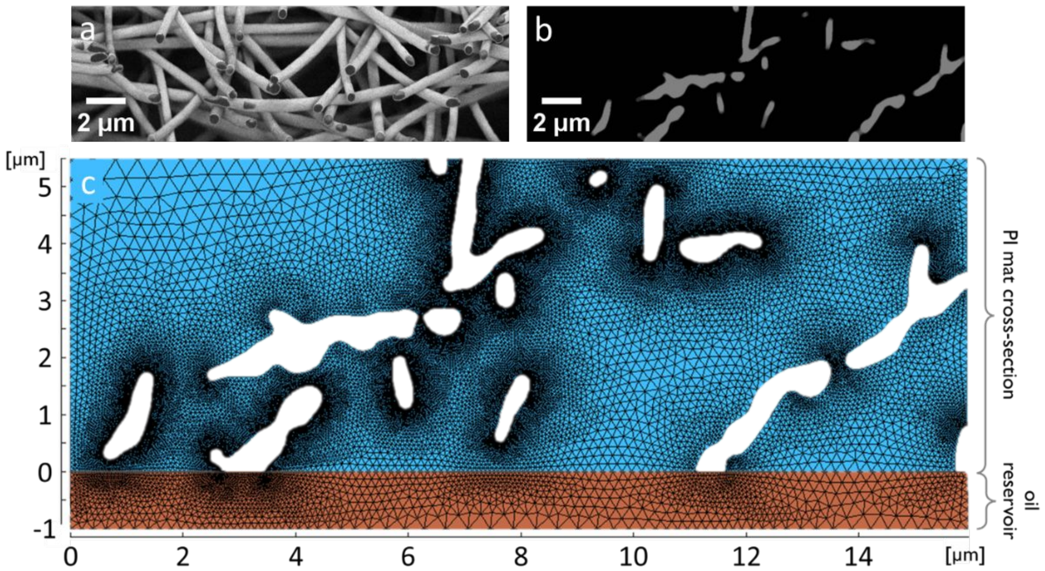

Figure S3. FIB-SEM image of PI mat cross-section, (b) post-processed image used for meshing, (c) computational domain for simulation of oil transport through PI mat.

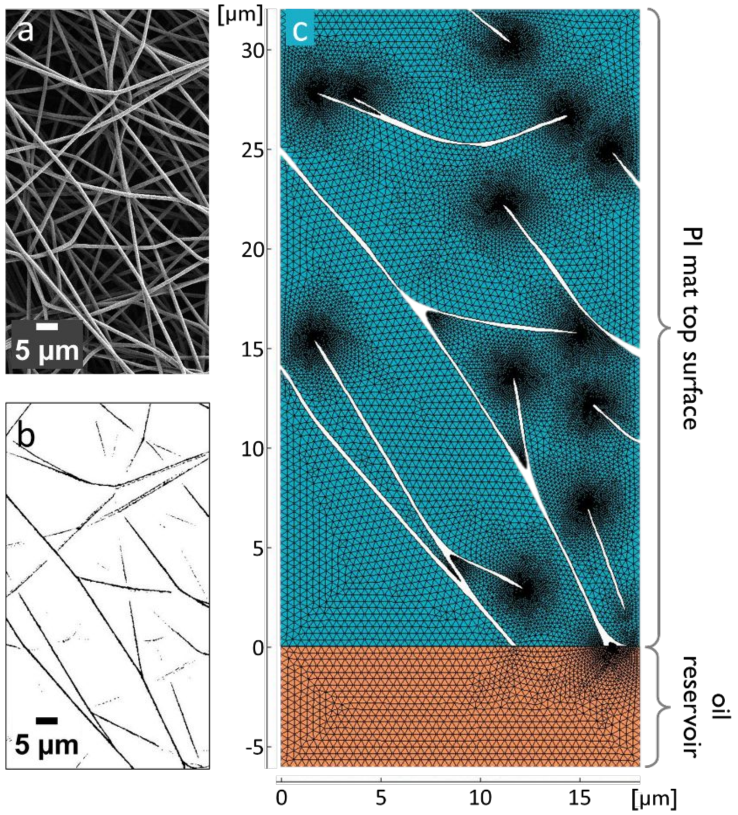

Figure S4. (a) SEM micrograph of PI mat surface, (b) post-processed image used for meshing, (c) computational domain for simulation of oil transport in the plane of the mat.

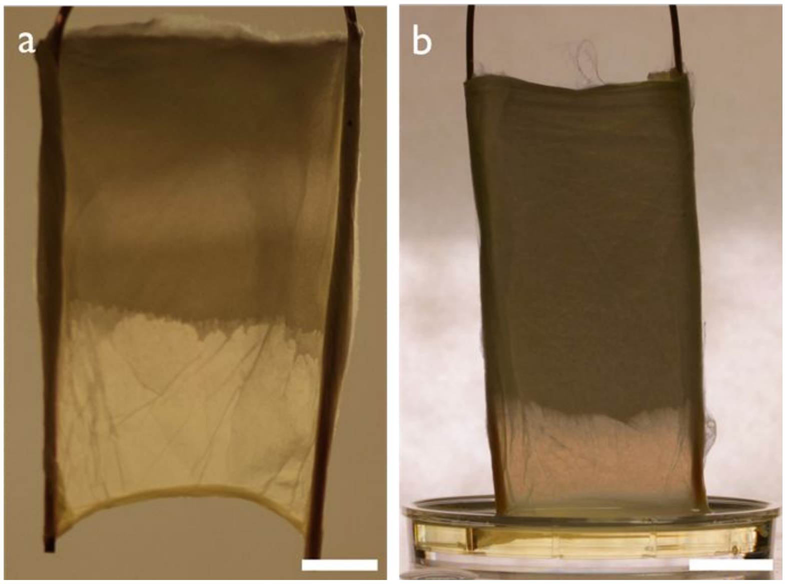

Figure S5. Experimental setup of capillary rise experiment: example pictures of oil capillary rise along PI mats (a) finite dose, (b) infinite dose. Scale bars are 5 mm.

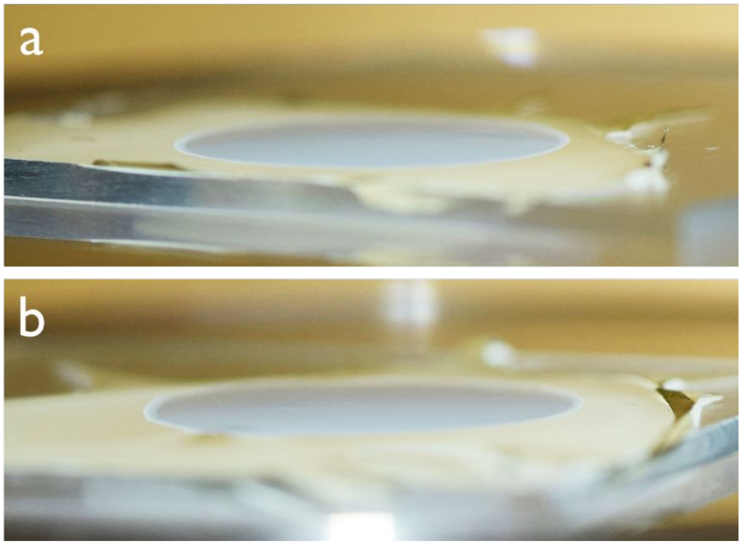

Figure S6. Wettability of PI films with (a) oil and (b) oil with 3% of cholesterol.

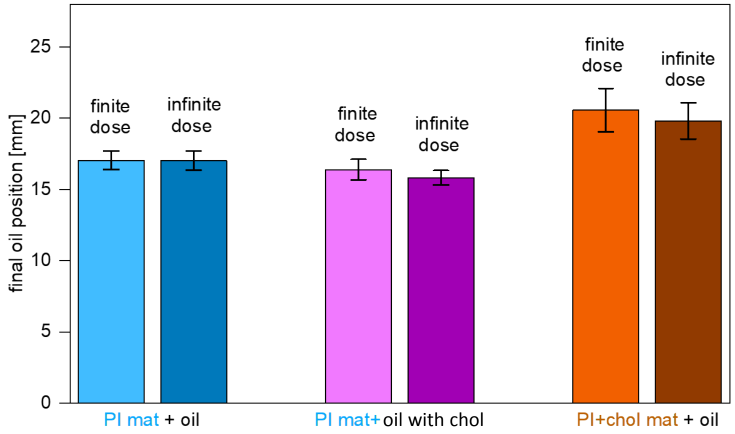

Figure S7. Final oil positions reached by oil or oil with cholesterol mixture in PI mats and PI mats with cholesterol after 1 h of capillary rise experiments.

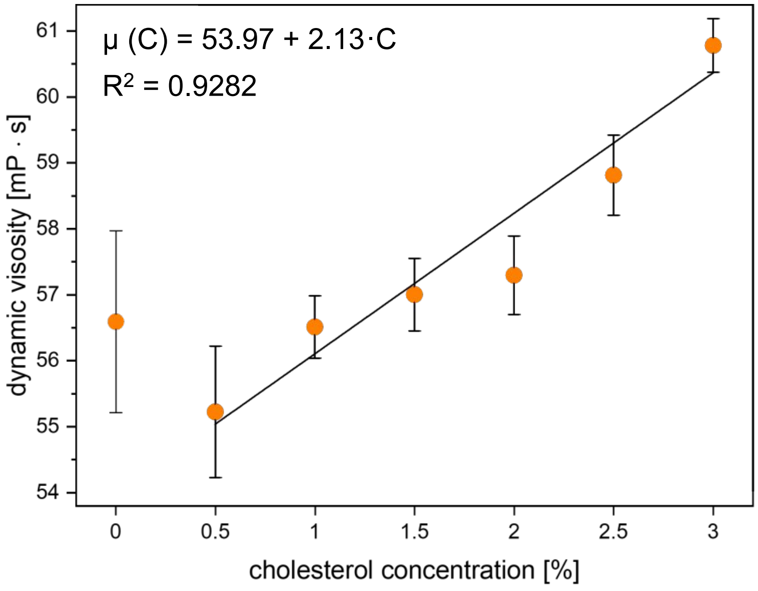

Figure S8. Dynamic viscosity of oil with cholesterol mixtures, $\boldsymbol{C}$ – mass concentration of cholesterol in blackcurrant seed oil.

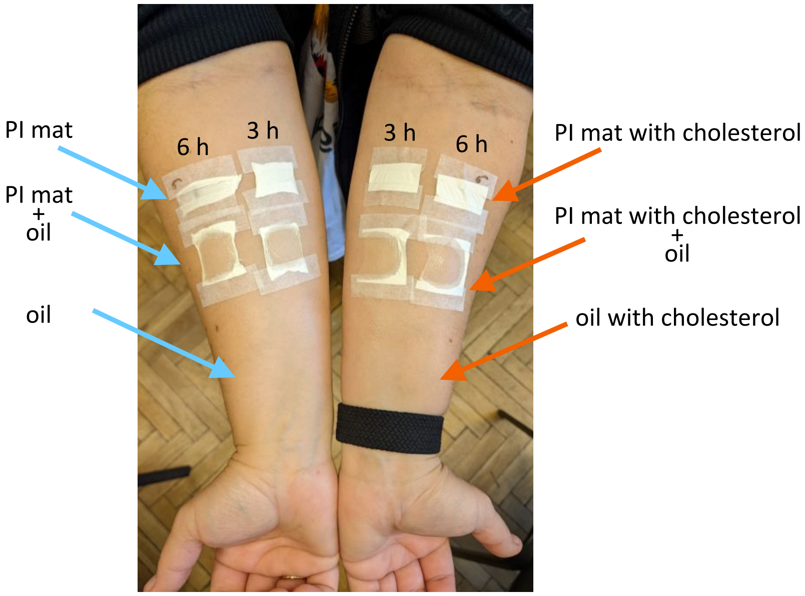

Figure S9. An example image from skin hydration tests after the application of patches and oils.

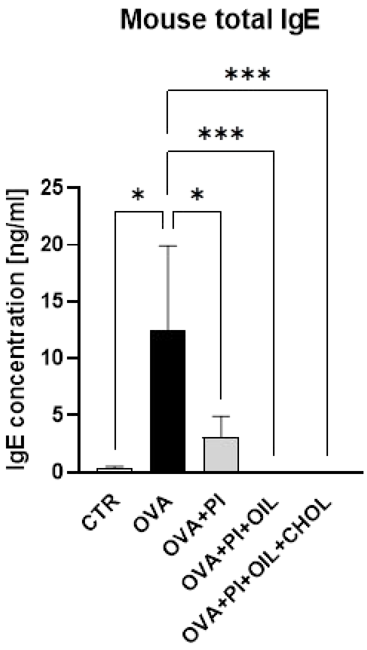

Figure S10. Total IgE levels in the mouse sera utilizing Chondrex Elisa KIT. The statistical analyses were performed using GraphPad software at the significance level *$p<0.05$, **$p<0.01$, ***$p<0.001$. The Shapiro-Wilk test was used to evaluate data normality. The Kruskal-Wallis test with post hoc analysis (Dunn's test) was applied to determine multiple comparisons.

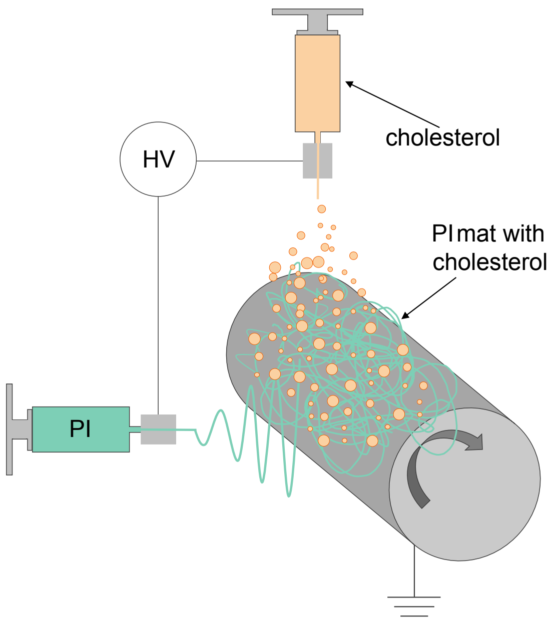

Figure S11. Schematics of PI mats with cholesterol fabrication by simultaneous PI electrospinning and cholesterol electrospraying.

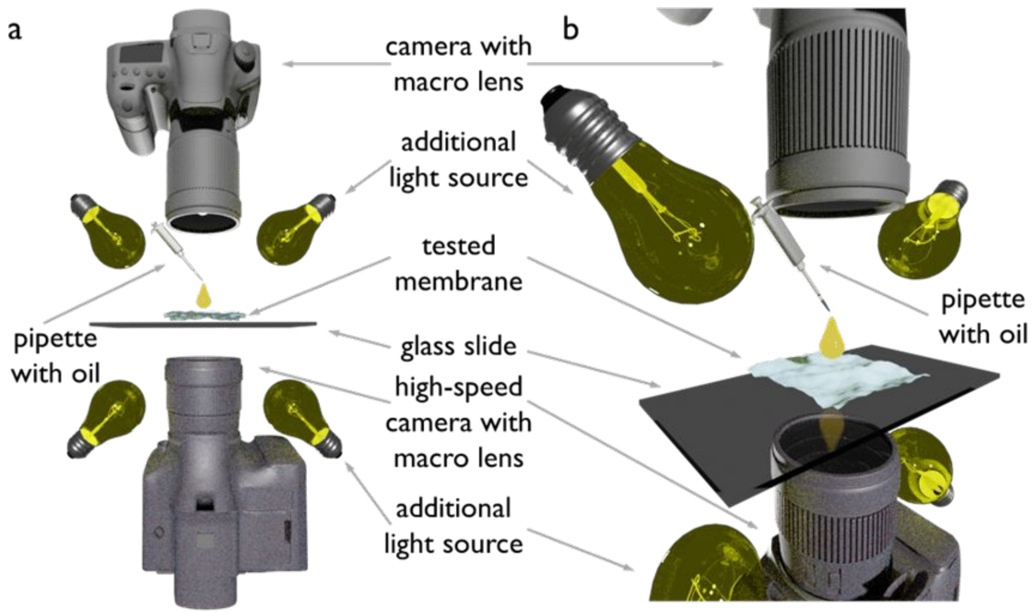

Figure S12. Experimental setup of oil in mats transport investigation: (a) front view, (b) side view.

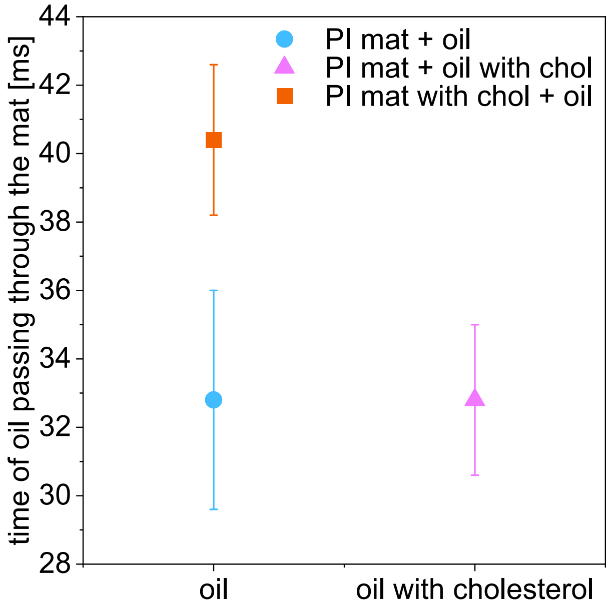

Figure S13. Time of oils passing through the mats – experimental data.

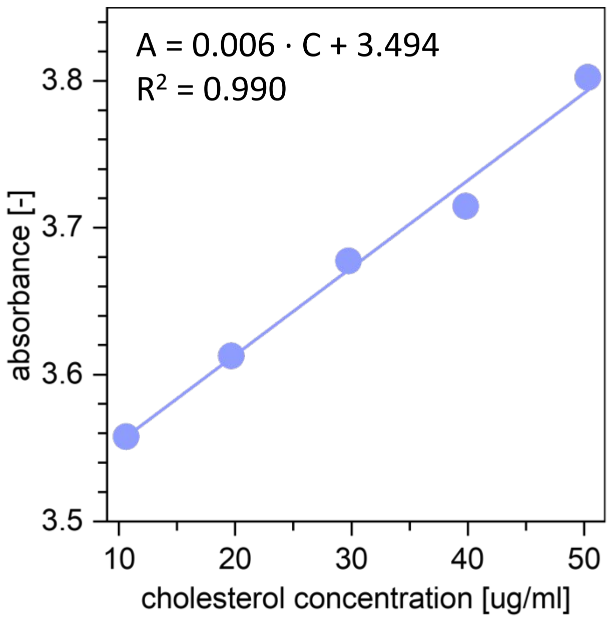

Figure S14. Calibration curve of cholesterol in blackcurrant seed oil, A – UV-vis absorbance, C – cholesterol concentration in blackcurrant seed oil [ug/ml].

Table S1. FTIR bands for raw cholesterol, electrosprayed cholesterol, PI mat, and PI with cholesterol mat.

band [cm^{-1}]	representation	raw cholesterol	electrosprayed cholesterol	PI mat	PI with cholesterol mat
720	imide ring deformation [2,3], rocking vibration of methylene chain [4]		✓	✓	✓
1055	represent ring deformation of cholesterol [5]	✓	✓		
1091-1096	represent ring deformation of cholesterol [5], OC–N–CO presence [3]			✓	✓
1360	represents bending CH_2 and CH_3 vibrations [6], symmetric carboxylate stretch [7]	✓	✓	✓	✓
1719-1720	symmetric C = O stretch [2,7], imide I antisymmetric stretch [8]		✓	✓	✓
2862-2864	symmetric stretching vibrations of CH_2 and CH_3 groups [5]	✓	✓		✓
2928-2930	asymmetric stretching vibrations of CH_2 and CH_3 groups [5]	✓	✓		✓

Table S2. Shape factor values from fitting the experimental data of capillary rise to Eq. 1.

	shape factor [-]		R^2	
dose	finite	infinite	finite	infinite
PI mat + oil	1.6155	1.4424	0.9924	0.7567
PI mat + oil with cholesterol	1.4887	1.3312	0.9963	0.9848
PI with cholesterol mat + oil	2.2593	1.9256	0.9866	0.9877

Table S3. Statistically significant differences for control mats from skin hydration tests and data presented in Figure 3b-d

	{1}	{2}	{3}	{4}
PI mat control 3 h				
PI mat control 6 h				
PI with cholesterol mat control 3 h				*
PI with cholesterol mat control 6 h			*	

Table S4. Statistically significant differences for PI mats and oil from skin hydration tests and data presented in Figure 3b-d

	{1}	{2}	{3}	{4}
PI mat + oil 3 h				
PI mat + oil 6 h			*	
oil 3 h		*		*
oil 6 h			*	

Table S5. Statistically significant differences for PI with cholesterol mats and oils from skin hydration test.

	{1}	{2}	{3}	{4}
PI with cholesterol mat + oil 3 h				*
PI with cholesterol mat + oil 6 h				*
oil + cholesterol 3 h				
oil + cholesterol 6 h	*	*		

SI References

(1) Sroczyk, E. A.; Berniak, K.; Jaszczur, M.; Stachewicz, U. Topical Electrospun Patches Loaded with Oil for Effective Gamma Linoleic Acid Transport and Skin Hydration towards Atopic Dermatitis Skincare. *Chem. Eng. J.* **2021**, *429* (September 2021), 132256. https://doi.org/10.1016/j.cej.2021.132256.

(2) Chern, Y. T. Low Dielectric Constant Polyimides Derived from Novel 1,6-Bis[4-(4-Aminophenoxy)Phenyl]Diamantane. *Macromolecules* **1998**, *31* (17), 5837–5844. https://doi.org/10.1021/ma970930b.

(3) Guzmán-Lucero, D.; Palomeque-Santiago, J. F.; Camacho-Zúñiga, C.; Ruiz-Treviño, F. A.; Guzmán, J.; Galicia-Aguilar, A.; Aguilar-Lugo, C. Gas Permeation Properties of Soluble Aromatic Polyimides Based on 4-Fluoro-4,4'-Diaminotriphenylmethane. *Materials (Basel).* **2015**, *8* (4), 1951–1965. https://doi.org/10.3390/ma8041951.

(4) Ishida, H.; Kamoto, R.; Uchida, S.; Ishitani, A.; Ozaki, Y.; Iriyama, K.; Tsukie, E.; Shibata, K.; Ishihara, F.; Kameda, H. Raman Microprobe and Fourier Transform-Infrared Microsampling Studies of the Microstructure of Gallstones. *Appl. Spectrosc.* **1987**, *41* (3), 407–412. https://doi.org/10.1366/0003702874448779.

(5) Gupta, U.; Singh, V.; Kumar, V.; Khajuria, Y. Spectroscopic Studies of Cholesterol: Fourier Transform Infra-Red and Vibrational Frequency Analysis. *Mater. Focus* **2014**, *3* (3), 211–217. https://doi.org/10.1166/mat.2014.1161.

(6) Raman, R.; Selvaraju, R. FTIR Spectroscopic Analysis of Human Gallstones. *Rom. J. Biophys* **2008**, *18* (4), 309–316.

(7) Diaham, S.; Locatelli, M.-L.; Khazak, R. BPDA-PDA Polyimide: Synthesis, Characterizations, Aging and Semiconductor Device Passivation. In *High Performance Polymers - Polyimides Based - From Chemistry to Applications*; 2012; Vol. 12, pp 15–36. https://doi.org/10.5772/53994.

(8) Pramoda, K. P.; Liu, S.; Chung, T. S. Thermal Imidization of the Precursor of a Liquid Crystalline Polyimide. *Macromol. Mater. Eng.* **2002**, *287* (12), 931–937. https://doi.org/10.1002/mame.200290027.
